# Store-operated Ca^2+^ entry in primary murine lung fibroblasts is independent of classical transient receptor potential (TRPC) channels and contributes to cell migration

**DOI:** 10.1038/s41598-020-63677-2

**Published:** 2020-04-22

**Authors:** Larissa Bendiks, Fabienne Geiger, Thomas Gudermann, Stefan Feske, Alexander Dietrich

**Affiliations:** 1 Walther Straub Institute of Pharmacology and Toxicology, Member of the German Center for Lung Research (DZL), Medical Faculty, LMU-Munich, Munich, Germany; 2 Department of Pathology, New York University School of Medicine, New York, NY 10016 USA

**Keywords:** Respiration, Cell migration, Cell signalling

## Abstract

Stromal interaction molecules (STIM1, 2) are acting as sensors for Ca^2+^ in intracellular stores and activate Orai channels at the plasma membrane for store-operated Ca^2+^ entry (SOCE), while classical transient receptor potential (TRPC) channel mediate receptor-operated Ca^2+^ entry (ROCE). Several reports, however, indicate a role for TRPC in SOCE in certain cell types. Here, we analyzed Ca^2+^ influx and cell function in TRPC1/6-deficient (TRPC1/6^−/−^) and STIM1/2- deficient (STIM1/2^ΔpmLF^) primary murine lung fibroblasts (pmLF). As expected, SOCE was decreased in STIM1/2- deficient pmLF and ROCE was decreased in TRPC1/6^−/−^ pmLF compared to control cells. By contrast, SOCE was not significantly different in TRPC1/6^−/−^ pmLF and ROCE was similar in STIM1/2-deficient pmLF compared to Wt cells. Most interestingly, cell proliferation, migration and nuclear localization of nuclear factor of activated T-cells (NFATc1 and c3) were decreased after ablation of STIM1/2 proteins in pmLF. In conclusion, TRPC1/6 channels are not involved in SOCE and STIM1/2 deficiency resulted in decreased cell proliferation and migration in pmLF.

## Introduction

Store-operated Ca^2+^ entry (SOCE) also named capacitive Ca^2+^ entry (CCE) was first described by J.W. Putney Jr. more than 30 years ago as depletion of intracellular Ca^2+^ stores which induces the opening of plasma membrane (PM) Ca^2+^ channels^1^. Since then, candidate proteins like classical transient receptor potential (TRPC) channels^2^ and mechanisms, e.g. coupling of TRPC proteins to inositol 1-4-5 trisphosphate (IP3) receptor channels in the endoplasmic reticulum^3,4^ for SOCE, were intensively discussed in the scientific community. In 2005 however, stromal interaction molecules (Stim in *Drosophila* and STIM1, STIM2 in humans) were identified as Ca^2+^ sensors in the ER directly regulating SOCE in two different large-scale screening approaches^5,6^. One year later, Ca^2+^ selective channels at the plasma membrane (Orai) were discovered^7–9^, which were responsible for Ca^2+^ release activated Ca^2+^ (CRAC) currents originally described in mast cells^10^. A molecular model was developed to support the concept that upon ER Ca^2+^ depletion STIM proteins homo-multimerize and translocate to ER-PM junctions^11,12^, where they recruit and gate Orai channels via direct interaction^13^. Ca^2+^ influx through Orai channels is important for cellular remodeling, e.g. in cardiovascular diseases^14^, and mutations in these channels are responsible for multiple channelopathies^15^. Irrespective of these events, TRP channels trigger Ca^2+^ influx in response to extracellular stimuli or receptor activation (receptor-operated Ca^2+^ influx, ROCE) independently of STIM and Orai^16^. Some labs, however, reported that TRPC channels also interact with STIM proteins^17^ and/or Orai channels^18^. Along these lines, TRPC channels like TRPC1 were invoked in SOCE in certain cells of salivary glands^19^ and pancreatic acini^20^, while in vascular smooth muscle cells TRPC1 channels work independently of SOCE^21^. The role of TRPC1 is even more confusing as the molecular architecture of native TRPC1 channels is still a matter of debate^22^. While all mammalian TRPC channels form homotetramers, the translocation of TRPC1 homotetramers to the plasma membrane and homomeric TRPC1 currents in native tissues were questioned^23^. In heteromeric TRPC channels TRPC1 appears to work as an ion channel regulator rather than an ion channel per se, because it modifies currents of homotetrameric TRPC5^24^ and reduces Ca^2+^ permeation of TRPC4/5/6/7 channels^25^. Therefore, the exact function of TRPC channels for SOCE or ROCE needs to be analyzed in each cell type independently.

In here, we set out to study the role of SOCE in primary murine lung fibroblasts (pmLF) using TRPC1/6- and STIM1/2-deficient fibroblasts in comparison to Wt control cells. SOCE was independent from TRPC1 and TRPC6 expression in pmLF but clearly dependent on STIM1/2 proteins. STIM1/2-deficiency reduced cell proliferation and migration as well as decreased nuclear levels of nuclear factor of activated T cells (NFATc1 and NFATc3) compared to control cells. Our data suggest an essential role of TRPC-independent SOCE in pmLF survival and cell migration.

## Materials and Methods

### Animals

*Stim1/2*^*flox/flox*^ mice were bred as previously described^26^. *Trpc1/6*^−/−^ mice were generated by crossing *Trpc1*^−/−^^21^ and *TRPC6*^−/−^ ^27^ mice. *Stim1/2*^*flox/flox*^ were crossed with *Trpc1/6*^−/−^ animals to gain *Stim1/2*^*flox/flox*^/*Trpc1/6*^−/−^ mice. All mice were on a C57BL/6J background. All animal experiments were approved by the governmental authorities and guidelines of the European Union (EU) were followed for the care and use of animals.

### Isolation and culture of primary murine lung fibroblasts

Primary murine lung fibroblasts (pmLF) were isolated as previously described for human lung fibroblasts^28^. Briefly, lungs of C57BL/6 mice were flushed through the right heart with sterile, cold PBS and excised. The lungs were dissected into pieces of 1–2 cm^2^ in size and digested by 1 mg/ml of Collagenase I (Biochrom, Cambridge, UK) at 37 °C for 2 h. Digested lung pieces were filtered through a nylon filter (pore size 70 μm; BD Falcon, Franklin Lakes, NJ, USA) and centrifuged for 5 min. Subsequently, the pellet was re-suspended in DMEM/F12 fibroblast culture medium (Lonza, Basel, Switzerland) supplemented with 20% fetal bovine serum (Invitrogen, Carlsbad, USA) as well as penicillin/streptomycin (Lonza, Basel, Switzerland) and normocin (InvivoGen, San Diego, USA) before finally plated on 10 cm cell culture dishes. Medium was changed after 2 days and cells were split after reaching a confluence of 80–90%. Only pmLF from passage 3–4 were used for the studies.

### Lentiviral infection of pmLF

PmLF from *Stim1/2*^*flox/flox*^ mice were infected by lentiviruses expressing Cre recombinase to obtain STIM1/2- deficient fibroblasts. Lentiviruses were produced as previously described^29^ based on the protocol for the amplification of second generation lentiviruses from the Tronolab (tronolab.epfl.ch). Lenti-X 293T cells (Clontec/Takara, Mountain View, USA) grown in DMEM medium (Lonza, Basel, Switzerland) supplemented with 10% fetal bovine serum (Invitrogen, Carlsbad, USA) as well as penicillin/streptomycin (Lonza, Basel, Switzerland) were transfected with pWPXL (carrying the gene of interest), pMD2G (encoding VSV G envelope protein) and pSPAX (encoding HIV-1 Gag, Pol, Tat and Revprotein) by calcium phosphate transfection. Supernatant containing virus was collected for two days. Virus solution was concentrated by using Peg-it solution (SBI, Mountain View, USA) and the pellet was re-suspended in cold PBS, aliquoted and stored at −80 °C. Successful virus production was verified by LentiX Go-stix (Clontec/Takara, Mountain View, USA). PmLF of the second passage were seeded at 1.5 × 10^5^ cells per well of a 6-well plate and infected by lentiviruses expressing Cre recombinase on the next day. Medium was changed the next morning and infected pmLF were used for experiments after 4–5 days. Excision of exons from *Stim1* and *Stim2* genes was monitored by genomic PCR.

### Genomic PCR

Genotyping of *Trpc1/6*^−/−^ and *Stim1/2*^*flox/flox*^ mice as well as STIM1/2^ΔpmLF^ fibroblasts was done as described^21,26,27^.

### Ca^2+^ imaging of intracellular Ca^2+^

STIM1/2^ΔpmLF^, TRPC1/6^−/−^, and TRPC1/6^−/−^ STIM1/2^ΔpmLF^ as well as control cells (Wt and Wt infected Cre recombinase expressing lentiviruses) were grown on 25 mm coverslips and loaded with Fura-2-AM (2 µM, Sigma, Taufkirchen, Germany) in 0.1% BSA in HEPES/HBSS buffer at 37 °C for 30 min. Coverslips were washed with HEPES/HBSS buffer and placed on a microscope in a low-volume recording chamber. To measure receptor-operated Ca^2+^ entry (ROCE) endothelin-1 (4 µM, Merck, Darmstadt, Germany) was applied in HBSS buffer with (2 mM) Ca^2+^ or in nominal Ca^2+^ free (0.5 mM EGTA) buffer after adding Ca^2+^ (2 mM). Store-operated Ca^2+^ entry (SOCE) was analyzed after depletion of internal Ca^2+^ stores by 1 µM thapsigargin (Sigma, Taufkirchen, Germany) in Ca^2+^ free HBSS solution containing 0.5 mM EGTA by adding extracellular Ca^2+^ (2 mM)^30^. An increase in intracellular Ca^2+^ ([Ca^2+^]_i_) was recorded using a Polychrome V monochromator (Till Photonics, Martinsried, Germany) and a 14-bit EMCCD camera (iXON3 885, Andor, Belfast, UK) coupled to an inverted microscope (IX71with an UPlanSApo 20×/0.85 oil immersion objective, Olympus, Hamburg, Germany) at 340 and 380 nm as described^30^.

### Quantitative reverse transcription (qRT)–PCR analysis

Total RNA from primary lung fibroblasts was isolated using the Invitrap Spin Universal RNA Mini Kit (Stratec, Berlin, Germany) according to the manufacturer’s protocol. First-strand cDNA was synthesized from the isolated total RNA using RevertAid RT containing reverse transcription polymerase (ThermoScientific, St. Leon-Rot, Germany) and random primer. MRNA expression of targeted genes in pmLF was analyzed by real time PCR as previously described^30^. Briefly, 10 pmol of each primer pair and 2 μl from the first strand synthesis were added to the reaction mixture consisting of 2x ABsolute QPCR SYBR Green Mix (ThermoScientific, St. Leon-Rot, Germany) and water. PCR was carried out in a light-cycler apparatus (Roche, Mannheim, Germany) using the following conditions: 15 min initial activation and 45 cycles of 12 s at 94 °C, 30 s at 50 °C, 30 s at 72 °C. Primer pairs (see Table 1) were used for the amplification of specific DNA-fragments from the first strand synthesis. Fluorescence intensities were recorded after an extension step at 72 °C after each cycle. Samples containing primer dimers were excluded by melting curve analysis and identification of the products were done by agarose gel electrophoresis. Crossing points were determined by the software program provided by the manufacturer. Relative gene expression was quantified using the formula: (2e(Crossing point reference gene − Crossing point X)) × 100 = % of the reference gene expression of the housekeeping gene (β-actin).

**Table 1:** Primer pairs used for amplification of quantitative RT-PCR fragments.

Target	Species	Forward primer (5′-3′)	Reverse primer (5′-3′)
STIM1	mouse	AAG CTT ATC AGC GTG GAG GA	CCT CAT CCA CAG TCC AGT TGT
STIM2	mouse	GAG GGC GCA GAG TGT GAG	TTT AGA GCC ATG CGG ACC T
Orai1	mouse	TAC TTA AGC CGC GCC AAG	ACT TCC ACC ATC GCT ACC A
Orai2	mouse	GGA CCT CAG CCC TCC TGT	GGG TAC TGG TAC TTG GTC TCC A
Orai3	mouse	CAC ATC TGC TCT GCT GTC G	GGT GGG TAT TCA TGA TCG TTC T
TRPC1	mouse	TGA ACT TAG TGC TGA CTT AAA GGA AC	CGG GCT AGC TCT TCA TAA TCA
TRPC3	mouse	TGG ATT GCA CCT TGT AGC AG	ACC CAG AAA GAT GAT GAA GGA G
TRPC4	mouse	GAT GAT ATT ACC GTG GGT CCT G	GAT TCC ACC AGT CAT GGA TGT
TRPC5	mouse	CTC TAC GCC ATC CGC AAG	TCA TCA GCG TGG GAA CCT
TRPC6	mouse	GCA GCT GTT CAG GAT GAA AAC	TTC AGC CCA TAT CAT GCC TA
TRPC7	mouse	AAT GGC GAT GTG AAC TTG C	CAG TTA GGG TGA GCA ACG AAC
β-actin	mouse	CTA AGG CCA ACC GTG AAA AG	ACC AGA GGC ATA CAG GGA CA

### Western blot analysis

Protein expression levels for STIM1 and STIM2 were determined by Western Blot analysis as previously described^30^. PmLF from cell culture dishes of 20 cm diameter were washed two times with cold PBS before 250 μl of lysis buffer (20 mM Tris-HCL, pH 7.5, 150 mM NaCl, 1% Nonidet P40, 0.5% sodium deoxycholate, 1% SDS, 5 mM EDTA) containing phosphatase und protease inhibitors (Roche, Mannheim, Germany) was applied for 60 min on ice. After centrifugation of the protein lysates at 5500 × g for 30 min at 4 °C protein concentration was quantified using a BCA-Assay (Pierce, Thermo Fisher, Schwerte, Germany) according to the manufacturer’s instructions. 6× Laemmli buffer (375 mM 4 × Tris/SDS buffer, pH 6.8, 48% glycerin, 6% SDS, 0,03% bromophenol blue and 9% β-mercaptoethanol) was added, the mixture incubated at 90 °C for 10 min and sonicated for 15 s. 10 μg protein of each sample was loaded on a 10% SDS gel. Protein separation was performed at room temperature using a current of 20 mA for 3–4 h. To transfer the proteins to a PVDF membrane a current of 20 mA was applied for 20 h at 4 °C. After transfer, the membrane was rinsed with 10 ml TBST for 5 min at room temperature. Transfer was checked using Ponceau solution (A2935 0500, AppliChem, Darmstadt, Germany). Blocking was performed for 1 h at room temperature using 10 ml blocking buffer (5% low fat milk in TBST). Each primary antibody was diluted in TBST containing 5% of blocking solution and applied over night at 4 °C. After washing with TBST three times for 10 min each, HRP-conjugated secondary antibody was applied for 2 h at room temperature. The membrane was washed with TBST three times, for 10 min each and incubated in SuperSignal West Femto chemiluminescent substrate (Thermo Scientific, Waltham, MA, USA). Chemiluminescence was detected by exposure of the filter in an Odyssey-Fc-unit (Licor, Lincoln, NE, USA). Used antibodies and dilutions: HRP-conjugated anti-β-actin antibody (Sigma A3854HRP, 1:10,000), anti-STIM1 (CellSignaling, #4916S, 1:1000), anti-STIM2 (CellSignaling, #4917S, 1:1000) and secondary anti-rabbit IgG peroxidase (POX)-antibody (Sigma A6154, 1:10000).

### Viability assay

Viability assays were performed by using the WST-1 Reagent (Roche, Mannheim, Germany) according to the manufacturer’s instructions. Tetrazolium salts like WST-1 are cleaved to colored formazan in viable cells, which can be measured by spectrophotometry. Cells were plated at a density of 2 × 10^4^ cells per ml per well of a 24-well plate and incubated at 37 °C and 5% CO_2_ overnight. WST-1 reagent was diluted 1:10 in pmLF medium before it was added to each well. After 3 hours of incubation absorbance of formazan was measured at a wavelength of 450 nm by spectrophotometry (Tecan, Switzerland). Cell free wells containing pmLF Medium plus WST-1 reagent served as background values.

### Proliferation assay

DNA synthesis of lung fibroblasts was assessed using the Click-iT 5-ethynyl-2′-deoxyuridine (EdU) Imaging Kit (ThermoScientific, St. Leon-Rot, Germany). In brief, 1.5 × 10^5^ pmLF per well of a 6 well plate were plated on coverslips overnight and were treated with 10 µM EdU for 3 hours on the next day. After washing cells with PBS and fixation in 3.7% formaldehyde for 10 min pmLF were treated according to the manufacturer’s protocol as previously described^29^. EdU is a thymidine analogue which gets incorporated into DNA during active DNA synthesis, if added to the culture medium^31^. After incorporation the ethynyl group of EdU covalently couples to a small fluorescent azide in a copper-dependent click reaction, which can be detected under a fluorescence microscope. To detect all cell nuclei, an additional staining with Hoechst 33342 (Life Technologies, Darmstadt, Germany) was performed. Stained cells were visualized by confocal imaging (LSM 880, Carl Zeiss) and stained nuclei were analyzed by the ImageJ software.

### Migration assay

Approximately 1.5 × 10^4^ cells per insert were seeded on a 3 well silicone insert with a 500 µm cell-free gap (ibidi GmbH, Martinsried, Germany) and grown at 37 °C and 5% CO_2_ overnight. Insert detachment created a defined cell-free gap. Images were taken 0, 4, 8, 12 and 24 h after releasing inserts. Migration was analyzed by measuring the remaining gap width by the ImageJ software in 3 pictures per time point and replicate.

### Isolation of nuclear fractions

Isolation of nuclear protein extracts from pmLF was performed with a Nuclear Extract Kit according to the manufacturer’s instructions (Active Motif, 40010, La Hulpe, Belgium) as described^30^. In brief, cells were first washed with PBS containing phosphatase inhibitors. Cytoplasmic protein fractions were collected by adding hypotonic lysis buffer and detergent, causing leakage of cytoplasmic proteins into the supernatant. After centrifugation (14.000 × g for 30 s) nuclear protein fractions were obtained by re-suspending pellets in detergent-free lysis buffer containing protease inhibitors. NFAT proteins were analyzed by Western Blotting as described above with the following modifications: 20 μl of each protein sample was loaded on a 7.5% SDS gel. Transfer of proteins to PVDF membrane was performed by an applied current of 360 mA at 4 °C for 1 h and 15 min. Unspecific binding sites were blocked in 5%BSA in TBST for one hour prior to incubation with the first antibody incubation overnight. All other steps were performed as described before. Antibodies used: Anti NFATc1 (mouse, SantaCruz Biotechnology, sc-7294, 1:500), anti-NFATc3 (mouse, SantaCruz Biotechnology, sc-7294, 1:500), anti-mouse IgG HRP- linked antibody (CellSignaling, #7076, Danvers, USA) as secondary antibody. Anti Lamin B1 (rabbit, ThermoScientific, PA5-19468, 1:5000) and secondary anti-rabbit IgG peroxidase-linked antibodies (goat, Sigma A6154, 1:10000) served as loading control. Protein bands were normalized to loading controls and quantified by an Odyssey-Fc unit (Licor, Lincoln, USA).

### Statistics

All statistical tests were performed using GraphPad Prism 7 (GraphPad Software, San Diego, USA). All Data were first tested for Gaussian distribution using the Shapiro-Wilk test. Gaussian distributed data were analyzed by t-tests or ordinary one-way ANOVA test. If Gaussian distribution was not assumed, nonparametric tests (Wilcoxon matched-pairs signed-rank test, Mann-Whitney U test or Kruskal-Wallis test) were used. Data are shown in means ± SEM. Significant differences are indicated by asterisks for P < 0.05 (*), 0.01 (**), 0.001 (***) and 0.0001 (****).

## Results

### Cre recombinase induced excision of exons in *Stim1* and *Stim2* genes in primary murine fibroblasts (pmLF) and resulting changes in mRNA and protein levels

**Figure 1:**
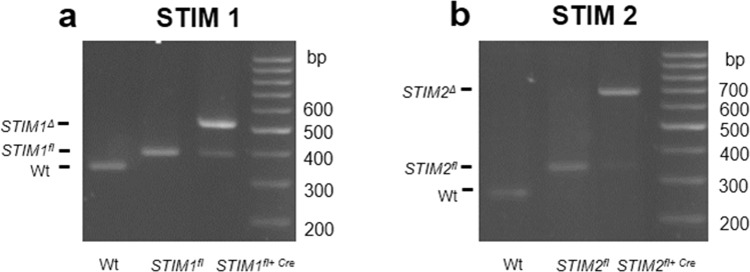
Representative images of PCR fragments obtained from genomic DNA of primary murine fibroblasts (pmLF) using gene specific primers separated by agarose gel electrophoresis (**a,b**). Wild-type (Wt), *Stim1/2*^*flox/flox*^ (*Stim1*^*fl*^, *Stim2*^*fl*^) as well as *Stim1/2*^*flox/flox*^ fibroblasts infected with lentiviruses expressing Cre recombinase (*Stim1*^*fl*+*Cre*^, *Stim2*^*fl*+*Cre*^) were analyzed. (**a**) DNA fragments amplified from Wt (Wt), *Stim1*^*flox/flox*^ (*Stim1*^*fl*^) or deleted *Stim1* (*Stim1*^Δ^) alleles are marked. (**b**) DNA fragments amplified from Wt (Wt), *Stim2*^*flox/flox*^ (*Stim2*^*fl*^) or deleted *Stim2* (*Stim2*^Δ^) alleles are marked.

To investigate the role of SOCE in pmLF we set out to delete exons in genes, which might be essential for SOCE. We first bred mice deficient for TRPC1 and TRPC6 to obtain *Trpc1/6*^−/−^ -double- deficient mice after a crossing over event on chromosome 9, where both genes are located. These mice are viable, fertile and have a normal life span. In clear contrast, STIM1/2 deficient mice die within a few weeks after birth^26^. To examine the effect of STIM deficiency in primary murine lung fibroblasts (pmLF), we therefore isolated these cells from mice with loxP flanked *Stim1* and *Stim2* genes^26^ and infected them with recombinant lentiviruses expressing Cre-recombinase. By genomic PCR, we detected DNA fragments corresponding to the deleted *Stim1* and *Stim2* genes after lentiviral infection, respectively (Fig. 1a,b).

**Figure 2:**
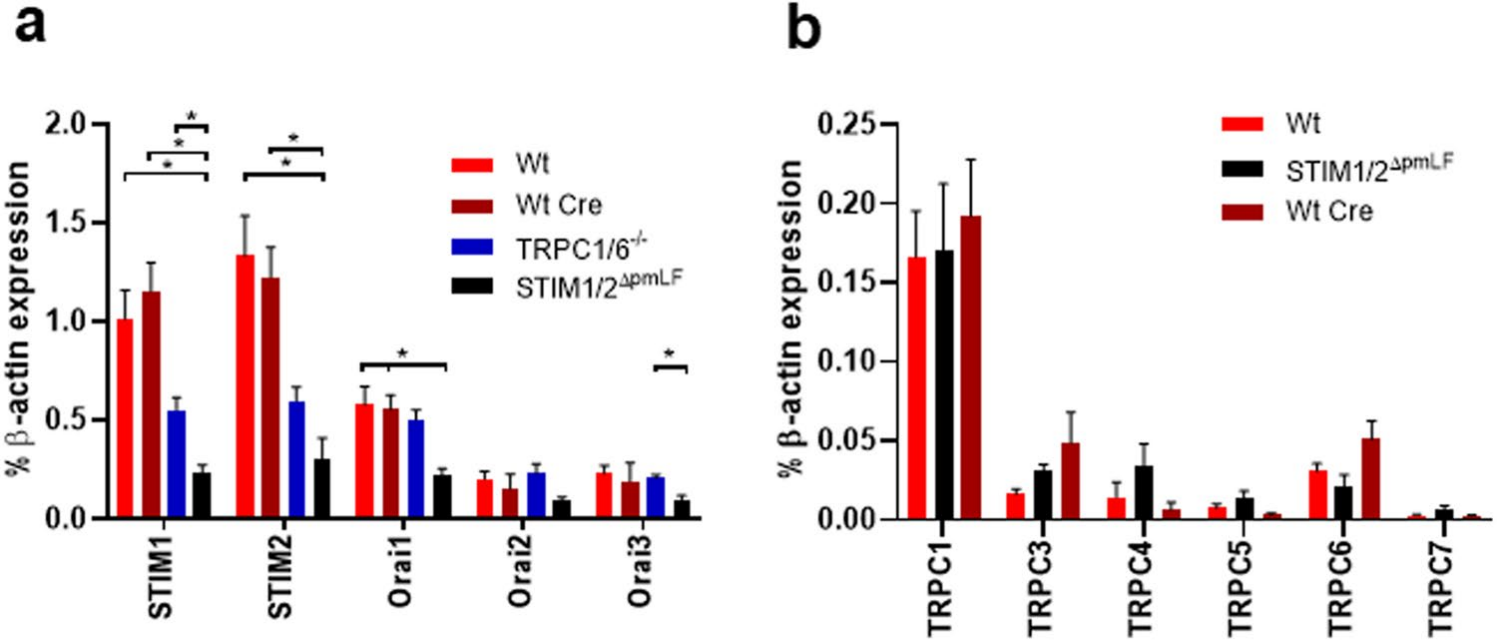
Quantification of STIM, Orai and TRPC mRNAs in primary murine lung fibroblasts (pmLF). Relative mRNA expression of STIM1 and STIM2 as well as Orai1-3 (**a**) or TRPC channels 1, 3–7 (**b**) in wild-type (Wt) pmLF, Wt cells infected with Cre recombinase expressing lentiviruses (Wt Cre), *STIM1/2*^*fl/fl*^ fibroblasts infected with Cre recombinase expressing lentiviruses (STIM1/2^ΔpmLF^) and TRPC1/6- (TRPC1/6^−/−^) deficient pmLF analyzed by quantitative RT-PCR. Columns show means +/− SEM (n > 3 mice, *P < 0.05, **P < 0.01, ***P < 0.001).

To test for any compensatory up- or down-regulation of STIM, Orai and TRPC1/6 mRNAs we quantified mRNA levels in STIM1/2 (STIM1/2^ΔpmLF^) and TRPC1/6-deficient (TRPC1/6^−/−^) fibroblasts in comparison to wild-type (Wt) and Wt cells infected with recombinant lentiviruses expressing Cre recombinase (Wt Cre). No up-regulation but significantly lower levels of STIM1-2 as well as Orai1-3 mRNAs were observed in STIM1/2^ΔpmLF^ fibroblasts (Fig. 2a,b). In TRPC1/6-deficient pmLF, we detected significantly decreased levels of TRPC1 and TRPC6 mRNA as expected. TRPC5 mRNA levels, although very low, were also significantly reduced in TRPC1/6−/− pmLF (Fig. S1 in Supplementary Information).

**Figure 3:**
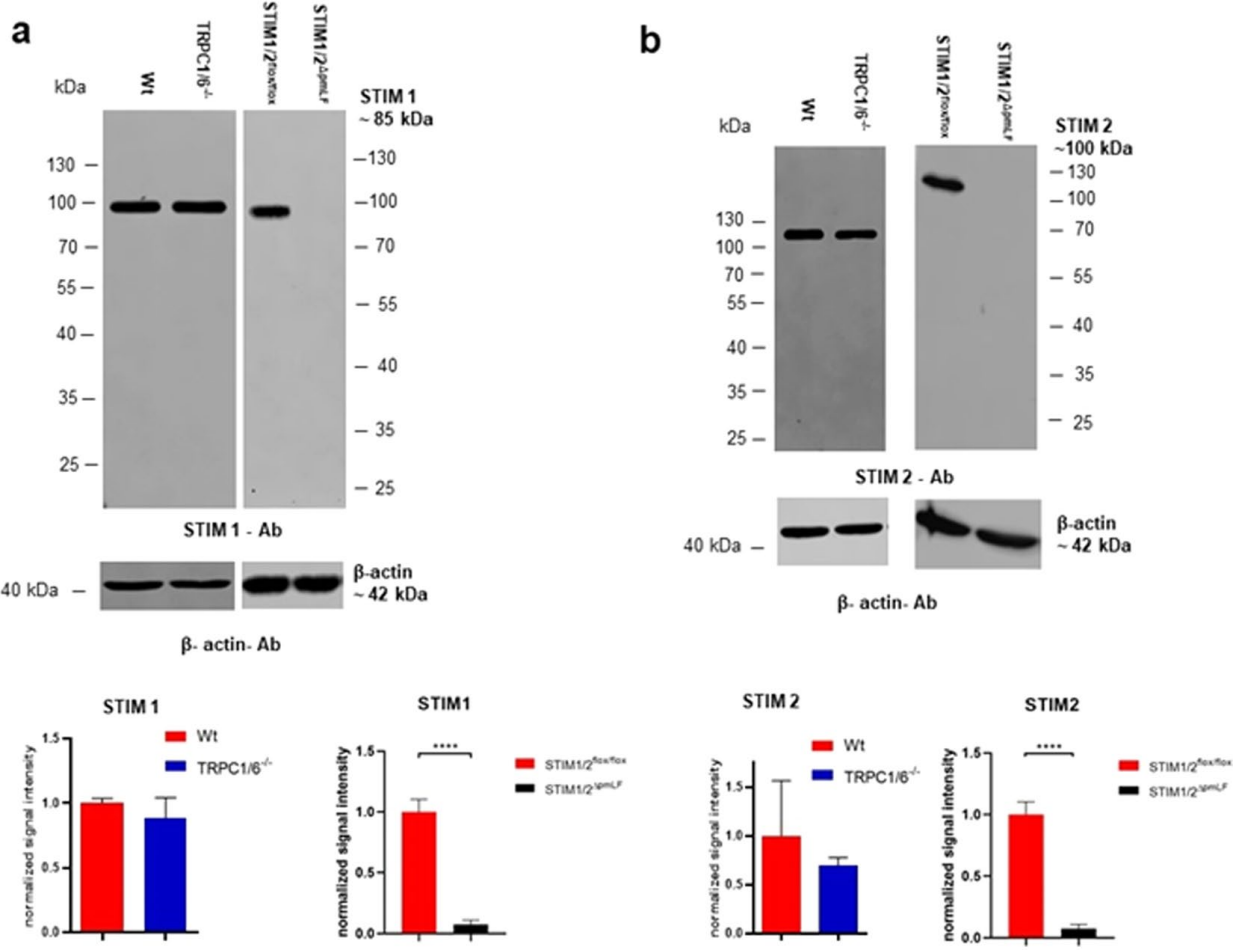
Quantification of STIM1 (**a**) and STIM2 (**b**) protein expression in cell lysates of of wildtype (Wt), TRPC1/6-deficient (TRPC1/6^−/−^), and STIM1/2-deficient (STIM1/2^ΔpmLF^) primary murine lung fibroblasts (pmLF). Expression of β-actin was used as loading control. Signals were normalized and quantified by using the LICOR software. Columns show means +/− SEM (n = 3 mice). Representative images from STIM1 and STIM2  immunoblots. Asterisks mark significant differences (****P < 0.0001).

In Western Blots STIM1 and STIM2 proteins were not detectable in STIM1/2^ΔpmLF^, but expressed in similar amounts in TRPC1/6^−/−^ pmLF as in Wt cells (Fig. 3a,b).

### Receptor-operated Ca^2+^ entry (ROCE) is decreased in TRPC1/6-deficient, but not significant different in STIM1/2-deficient fibroblasts

**Figure 4:**
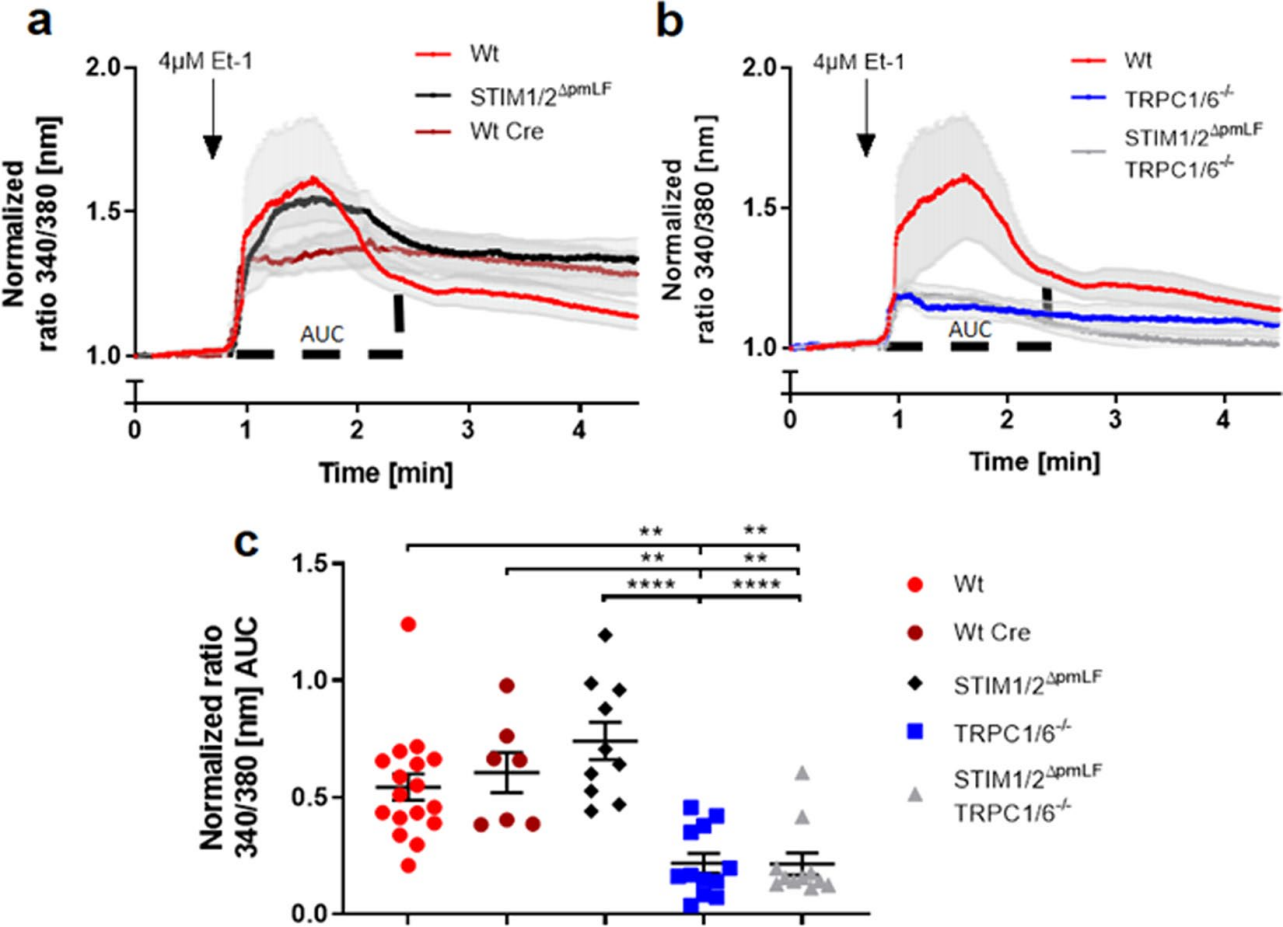
Receptor-operated Ca^2+^ entry (ROCE) induced by application of endothelin-1 (Et-1) in TRPC1/6- (TRPC1/6^−/−^) (**a**,**c**), STIM1/2− (STIM1/2^ΔpmLF^) (**b**,**c**) and STIM1/2−TRPC1/6− (TRPC1/6^−/−^ STIM1/2^ΔpmLF^) deficient primary murine lung fibroblasts (pmLF) (**a,c**). Wild-type pmLF infected with recombinant lentiviruses expressing Cre recombinase (Wt Cre) served as controls. Fura-2-loaded pmLF were stimulated with 4 µM Et-1 in Ca^2+^ containing buffer to generate ROCE. Intracellular Ca^2+^ levels ([Ca^2+^]_i_) were quantified by analysis of fluorescence ratios at excitation wavelengths of 340 and 380 nm (ratio 340/380 nm) and normalized to initial values. Lines represent calculated means and light grey areas indicate standard error of the mean (SEM) of more than three independent experiments of at least three mice. Calculation of the areas under the curves (AUC) in Fig. 4a,b was used to quantify ROCE (**c**). One single dot represents the mean of at least 20 cells from one cell isolation. Asterisks mark significant differences from left to right (n > 3 mice, **P < 0.01, ***P < 0.001, ****P < 0.0001) between ratios of deficient cells compared to control cells.

To quantify ROCE in primary murine lung fibroblasts (pmLF), endothelin 1 (Et-1) was used to activate Gq protein-coupled endothelin receptors. Stimulation of Phospholipases C-β by Gα_q_-subunits resulted in cleavage of phosphatidylinositol 4,5-bisphosphate (PIP2) and generation of diacylglycerol (DAG), which activates TRPC6 channels^32^. ROCE was quantified by analyzing Ca^2+^ transients measured fluorometrically at wavelengths of 340 and 380 nm (Fig. 4a,b) and calculating areas under the curve (AUC) (Fig. 4c) after adding Et-1 to pmLF. Both values were significantly decreased in TRPC1/6-deficient but not in STIM1/2-deficient pmLF compared to control cells (Fig. 4a–c). We also performed recalcification experiments after application of Et-1 in Ca^2+^ free buffer (Fig. S2 in Supplementary Information). While there is no difference in cytoplasmic Ca^2+^ levels after the release of Ca^2+^ from the internal stores following IP_3_ production, Ca^2+^ influx from the extracellular medium is reduced in TRPC1/6^−/−^ fibroblasts compared to Wt cells.

### Store-operated Ca^2+^ entry (SOCE) is reduced in STIM1/2-deficient, but not in TRPC1/6-deficient primary murine lung fibroblasts (pmLF)

**Figure 5:**
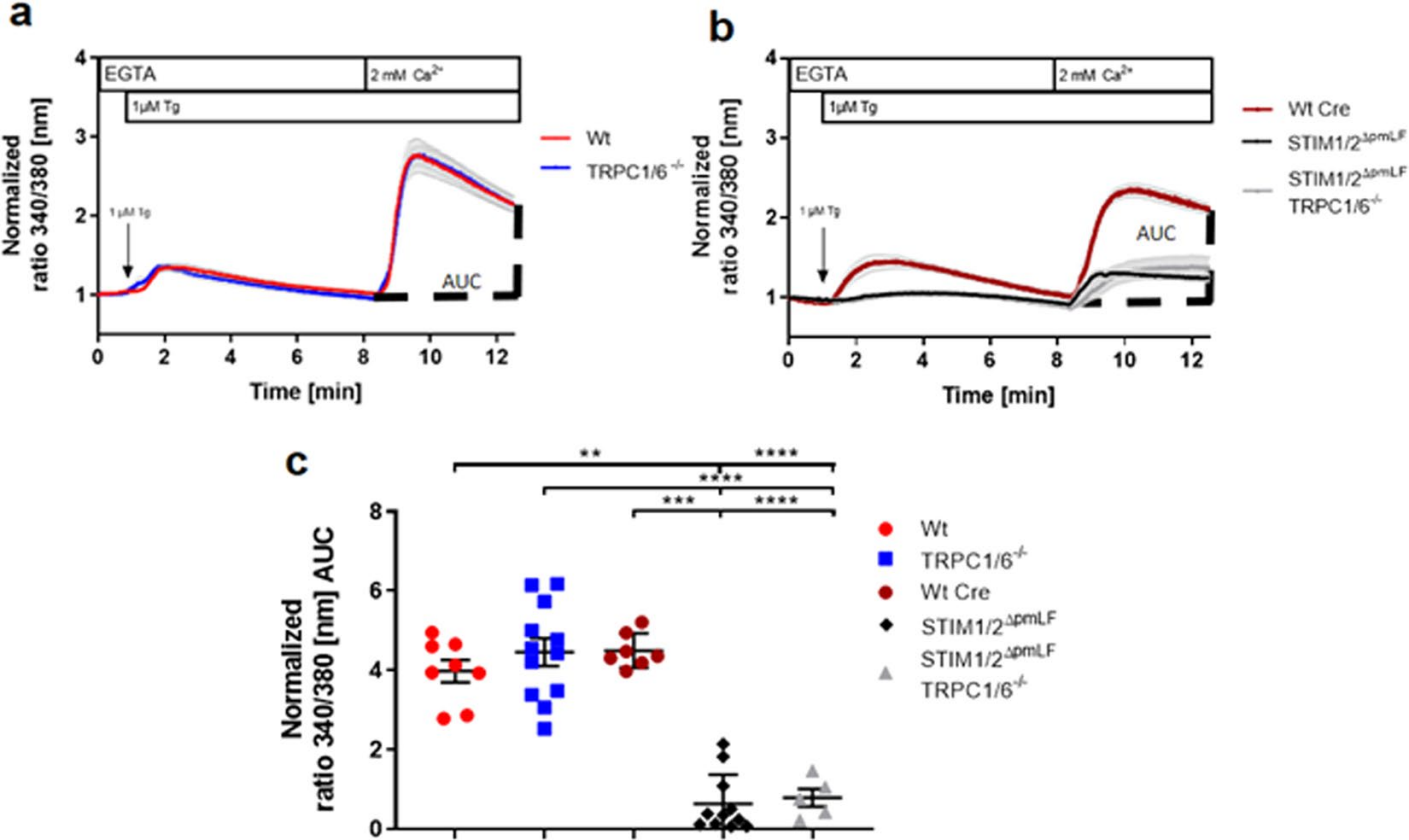
Store-operated Ca^2+^ entry (SOCE) induced by application of thapsigargin in Ca^2+^-free buffer and subsequent readdition of extracellular Ca^2+^ in TRPC1/6− (TRPC1/6^−/−^) (**a**,**c**), STIM1/2− (STIM1/2^ΔpmLF^) (**b**,**c**) and STIM1/2−TRPC1/6− (TRPC1/6^−/−^ STIM1/2^ΔpmLF^) deficient primary murine pulmonary fibroblasts (pmLF) (**a**,**c**). (**a**,**b**) Wild-type fibroblasts infected with recombinant lentiviruses expressing Cre recombinase (Wt Cre) served as controls. Internal Ca^2+^ stores of fura-2-loaded pmLF were emptied by application of thapsigargin followed by recalcification. Intracellular Ca^2+^ levels ([Ca^2+^]_i_) were quantified by analysis of fluorescence ratios at excitation wavelengths of 340 and 380 nm (ratio 340/380 nm) and normalized to initial values. Lines represent calculated means and light grey areas indicate standard error of the mean (SEM) of more than three independent experiments of at least three mice. Calculation of the areas under the curves (AUC) in Fig. 6a,b was used to quantify SOCE (**c**). One single dot represents the mean of at least 20 cells from one cell isolation. Asterisks mark from left to right significant differences (*P < 0.05, **P < 0.01, ***P < 0.001, ****P < 0.0001) between ratios of deficient cells compared to control cells.

SOCE was induced in pmLF by emptying internal Ca^2+^ stores after application of thapsigargin in Ca^2+^ free buffer containing the Ca^2+^ chelator EGTA and subsequent readdition of extracellular Ca^2+^. While TRPC1/6-deficient fibroblasts showed no differences, SOCE in STIM1/2-deficient cells was significantly reduced comparing peak levels and areas under the curve (AUC) (Fig. 5a–c). Ablation of all four proteins STIM1/2 and TRPC1/6 did not further reduce SOCE. Therefore, SOCE is exclusively regulated by STIM1/2 proteins and Orai channels in primary murine fibroblasts and not dependent on TRPC1 and TRPC6.

### STIM1/2 deficiency reduces cell proliferation, migration and nuclear localization of NFAT transcription factors in fibroblasts

**Figure 6:**
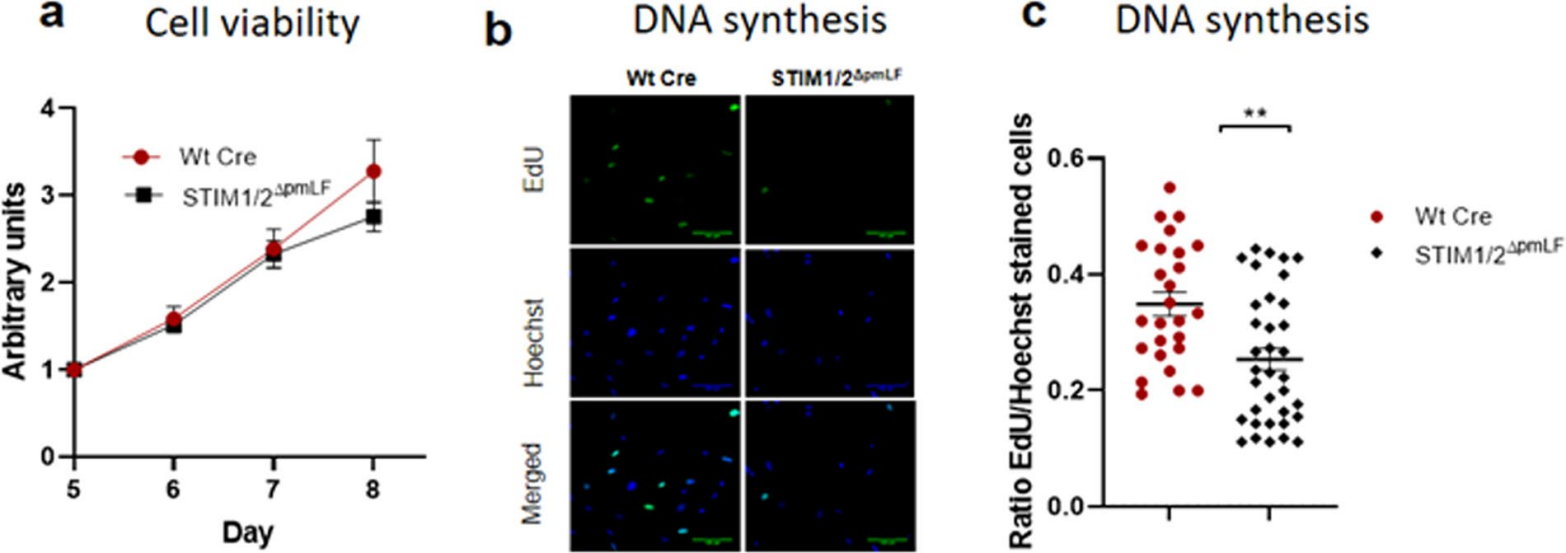
Cell viability (**a**) and DNA synthesis (**b**,**c**) quantified in STIM1/2- deficient primary murine lung fibroblasts (pmLF) compared to control cells. (**a**) Viability was analyzed in wild type pmLF infected with recombinant lentiviruses expressing Cre recombinase (Wt Cre) as well as STIM1/2- deficient pmLF using a WST-assay 5–8 days after infection. (**b**) Wild type pmLF infected with recombinant lentiviruses expressing Cre recombinase (Wt Cre) as well as STIM1/2- deficient pmLF were incubated with EdU (5-ethynyl-2′-deoxyuridine) for 4 hours and fixed cells were stained with cross- linked fluorescent azide. Nuclei staining was performed by Hoechst dye. (**c**) Individual values and means +/− SEM of EdU/Hoechst ratios were plotted . Asterisks mark significant differences (n > 3 mice, **P < 0.01) between ratios of STIM1/2-deficient cells compared to control cells.

To understand the general role of SOCE in cell function of pmLF, we quantified cell viability using a WST assay in STIM1/2- deficient fibroblasts in comparison to control cells. Cell viability was not impaired by STIM1/2 deficiency 5 to 8 days after infection with recombinant lentiviruses expressing Cre recombinase in comparison to infected and non-infected Wt pmLF (Fig. 6a). In contrast to these results. DNA synthesis as a measure of cell proliferation was significantly reduced in STIM1/2- deficient fibroblasts in comparison to Wt cells infected with recombinant lentiviruses expressing Cre recombinase (Fig. 6b,c).

**Figure 7:**
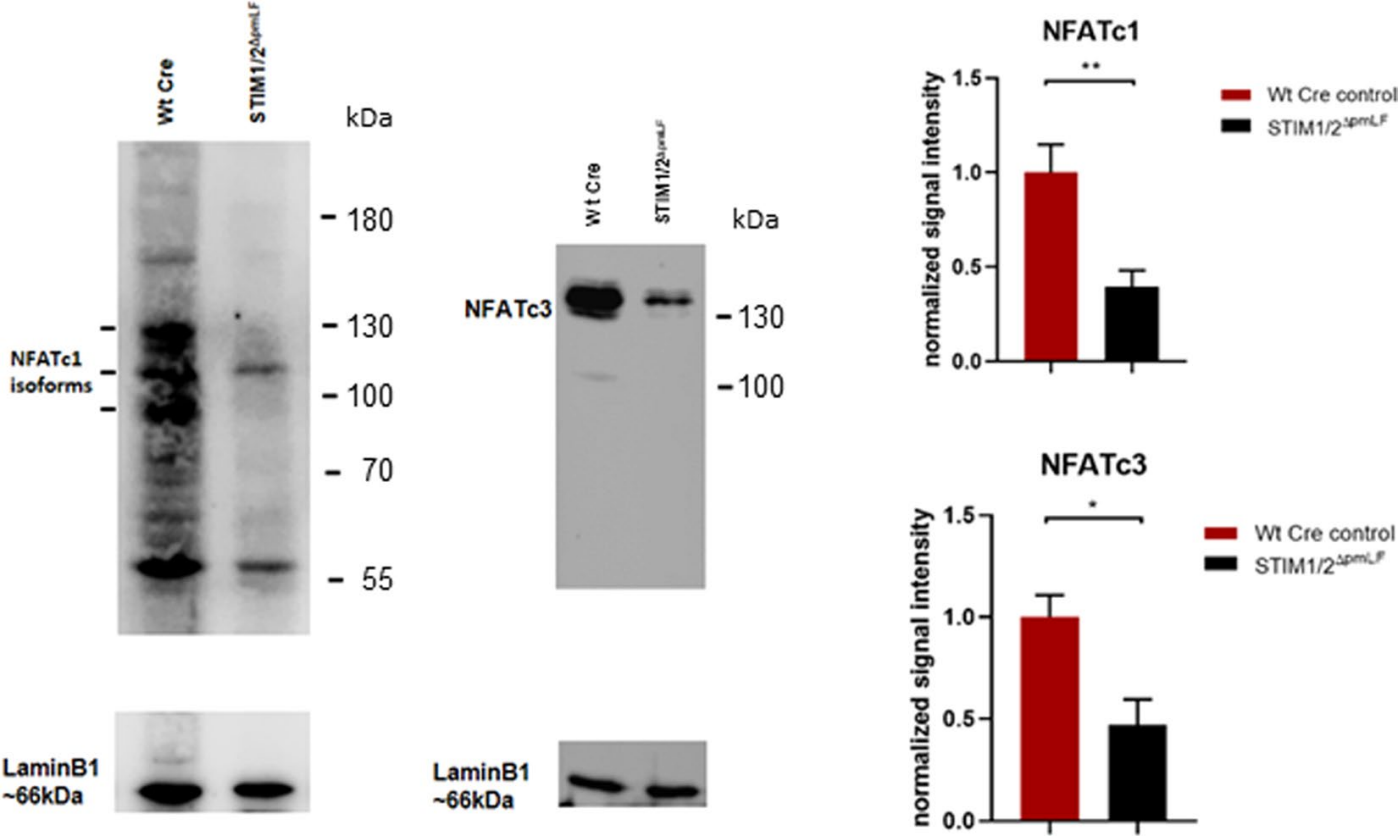
Quantification of nuclear NFATc levels in STIM1/2- deficient pmLF (STIM1/2^ΔpmLF^) and wild-type cells infected with recombinant lentiviruses expressing Cre recombinase as control cells (Wt Cre). Representative Western Blots showing NFATc1 (left panel) and NFATc3 (right panel) isoforms in nuclear extracts from STIM1/2^ΔpmLF^ and Wt Cre cells. Summary of quantitative analysis of nuclear NFAT levels of the c1 (upper bar graph) and c3 (lower bar graph) isoforms. Columns show calculated means +/− SEM. Asterisks mark significant differences (n > 3 mice, *P < 0.05, **P < 0.001) between ratios of STIM1/2^ΔpmLF^ cells compared to Wt Cre control cells.

To analyze SOCE on a molecular level in pmLF, we quantified nuclear levels of the Ca^2+^-dependent transcription factor nuclear factor of activated T cells (NFAT). Both isoforms NFATc1 and NFATc3 were significantly reduced in nuclear extracts of STIM1/2- deficient pmLF compared to Wt pmLF infected with recombinant lentiviruses expressing Cre recombinase as control cells (Fig. 7).

**Figure 8:**
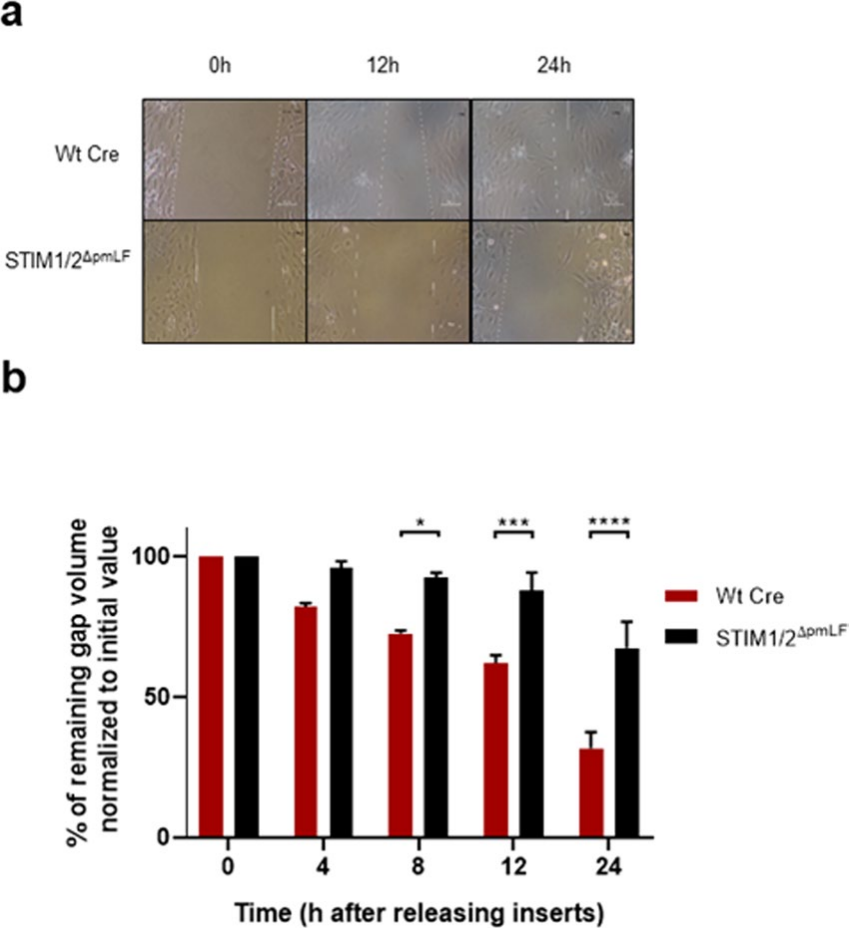
Migration of STIM1/2- deficient primary murine lung fibroblasts (pmLF) and control cells (**a**,**b**). (**a**) Images from a representative migration assay with STIM1/2- deficient pmLF compared to Wt cells infected with recombinant lentiviruses expressing Cre recombinase (Wt Cre) after removing inserts. (**b**) Summary of remaining gap values normalized to initial values quantified in migration assays of STIM1/2- deficient pmLF compared to Wt cells infected with recombinant lentiviruses expressing Cre recombinase (Wt Cre) after removing inserts at 0, 4, 8, 12 and 24 h. Data represent means ± SEM from 3 independent cell preparations of 5 mice each. Asterisks mark significant differences (*P < 0.05, **P < 0.01, ***P < 0.001, ****P < 0.0001) between ratios of STIM1/2-deficient cells compared to control cells.

An important function during repair processes by pmLF is cell migration. To ask whether SOCE may have a role in migration of pmLF, we quantified gap closure times of migrating STIM1/2- deficient and control pmLF. It took STIM1/2- deficient pmLF significantly longer to close a defined gap in a confluent cell layer compared to control cells (Wt and Wt Cre) (Fig. 8).

## Discussion

To dissect the molecular correlate of ROCE and SOCE in pmLF we deleted essential genes involved in one or presumably both processes in pmLF. In a former publication, we identified TRPC1 as the predominantly expressed TRPC channel in pmLF, while TRPC6 is up-regulated in TGF-β1 induced fibroblast to myofibroblast differentiation^30^. Three members of this TRPC family namely TRPC3, TRPC6 and TRPC7 are activated by diacylglycerol (DAG), which is produced after ligand binding to G protein-coupled receptors (GPCR) and subsequent cleavage of phosphatidylinositol 4,5-bisphosphate (PIP2) by phospholipase-C isozymes^32,33^. These so called DAG-sensitive channels are mainly expressed in brain, endothelial and smooth muscle cells of the vasculature and mediate ROCE (reviewed in^34^). Recent findings indicate that Na^+^/H^+^ exchanger regulatory factors dynamically determine the DAG sensitivity of TRPC4 and TRPC5 channels^35^ and that TRPC1 proteins work as channel regulators in heteromeric complexes with all other six channels of the TRPC family^25^. Therefore, all TRPC channels are responsible for ROCE and global ablation of TRPC1 and TRPC6 proteins is sufficient to reduce intracellular Ca^2+^ levels during ROCE in pmLF from TRPC1/6- deficient mice (Fig. 4).

Activation of ubiquitously expressed Orai channels in the plasma membrane is exclusively dependent on the multimerization of the ER Ca^2+^ sensors STIM1 and 2 after detecting reduced Ca^2+^ levels after store depletion (summarized in^36^). As global deletion of STIM1 and 2 proteins induces early death in the corresponding gene-deficient mouse models^26^, we choose a different approach and isolated pmLF from STIM1/2 floxed  mouse models, which carry loxP sites downstream and upstream of exons essential for protein function. Infection of these cells with recombinant lentiviruses expressing Cre recombinase efficiently deletes floxed exons in both genes (Fig. 1) resulting in an almost complete absence of both proteins (Fig. 3). No compensatory up-regulation of mRNAs for Orai and TRPC channels (Fig. 2) was detected. On a protein level, TRPC1/6- deficient pmLF expressed similar amounts of STIM1/2 proteins (Fig. 3) in comparison to Wt cells and no changes in SOCE were observed (Fig. 5a). In contrast, complete ablation of STIM1/2 proteins in STIM1/2^ΔpmLF^ significantly reduced SOCE (Fig. 5b), but had no significant effect on ROCE induced by activation of endothelin receptors by endothelin 1 (Et-1) (Fig. 4b). ROCE, however, was significantly reduced in TRPC1/6- deficient cells (Fig. 4a) confirming the hypothesis that TRPC1 and -6 channels are responsible for receptor-dependent Ca^2+^ influx in these cells.

Several reports indicate TRPC3-mediated SOCE in pancreatic acini^37^ and TRPC1-mediated SOCE in salivary gland cells^19^, which may be due to STIM1/2^17,38,39^ or Orai1/2/3^18,40,41^ interactions with TRPC channels in these cells (reviewed in^42,43^). In pmLF however, we were not able to detect any difference in SOCE in TRPC1/6- deficient cells compared to control cells (Fig. 5a,c), while STIM1/2- deficient pmLF showed significantly decreased levels (Fig. 5b,c). Therefore, we conclude that SOCE and ROCE in pmLF are mediated by different entirely independent molecular correlates in pmLF as already described in transiently transfected HEK293 cells^16^.

Next, we analyzed the role of SOCE in basal cell functions of pmLF. While metabolic activity indicative of cell viability was not changed in STIM1/2- deficient compared to lentivirus infected Wt cells (Fig. 6a), quantification of DNA synthesis as a marker for cell proliferation was decreased in STIM1/2- deficient cells compared to Wt cells infected with lentiviruses (Fig. 6b,c). The role of SOCE in cell proliferation has already been demonstrated in many other cell types (reviewed in^44^) and especially in cancer cells^45^. The nuclear translocation of NFATc transcription factors depends on increases in the intracellular Ca^2+^ concentration, which makes NFATc a preferred target for SOCE induced changes in cell function^46^. Accordingly, we identified lower levels of nuclear NFATc1 and 3 levels in STIM1/2- deficient pmLF compared to lentivirus infected Wt control cells emphasizing an important role of SOCE in Ca^2+^-induced mRNA transcription of pmLF (Fig. 7). Similar results were obtained for NFATc3 in arterial smooth muscle cells^47^. We also quantified cell migration as an essential function of pmLF during repair processes in the lung and detected significant longer gap closure times in STIM1/2- deficient pmLF compared to lentivirus infected control cells (Fig. 8). The role of SOCE in cell migration was intensively studied in cancer cells (reviewed in^48^), where intracellular Ca^2+^ influx through STIM-Orai interaction affects focal adhesion turnover as a critical step in the migration mechanism^49^. A similar mechanism may be important in migration of pmLF and needs to be further analyzed.

In summary, we were able to show for the first time that SOCE, which is exclusively induced by STIM1/2 proteins in the ER of pmLF, is not dependent on TRPC1 and TRPC6, the predominantly expressed TRP channels in pmLF. Therefore, an interaction of STIM proteins  and/or Orai channels with TRPC channels in these cells to mediate SOCE is unlikely. SOCE contributes to cell proliferation and migration as well as nuclear localization of nuclear factor of activated T-cells (NFATc1 and c3) in pmLF. Therefore, TRPC6 channels, which are also important for cellular functions of pmLF when differentiated to myofibroblasts by TGF-β1^30^, work independently of STIM1/2 proteins and Orai channels in this cell type.

## Supplementary information

Supplementary Information.

Supplementary Information2.
