# Supplementary material for: Store-operated Ca2+ entry in primary murine lung fibroblasts is independent of classical transient receptor potential (TRPC) channels and contributes to cell migration
Source: Sci Rep. 2020 Apr 22;10:6812. doi: 10.1038/s41598-020-63677-2 (PMC7176639; doi:10.1038/s41598-020-63677-2)
Supplement: Supplementary file 1 — Supplementary Information. [file 41598_2020_63677_MOESM1_ESM.pdf]

Store-operated Ca^{2+} entry in primary murine lung fibroblasts is independent of classical transient receptor channels and contributes to cell migration

Larissa Bendiks[1], Thomas Gudermann[1], Stefan Feske[2] & Alexander Dietrich[1*]

[1] Walther Straub Institute of Pharmacology and Toxicology, Member of the German Center for Lung Research (DZL), Medical Faculty, LMU-Munich, Munich Germany
[2] Department of Pathology, New York University School of Medicine, New York, NY, 10016, USA.
* email: alexander.dietrich@lrz.uni-muenchen.de

Supplementary Information

Contents

Figure S1: : Relative mRNA amounts of TRPC channels (TRPC1, 3–6) in WT and TRPC6−/− PMLFs analyzed by quantitative RT-PCR

Figure S2: Receptor-operated Ca^{2+} entry (ROCE) induced by application of endothelin-1 (Et-1) in Ca^{2+} free medium and after recalcification in TRPC1/6- (TRPC1/6$^{-/-}$) deficient primary murine lung fibroblasts (pmLF).

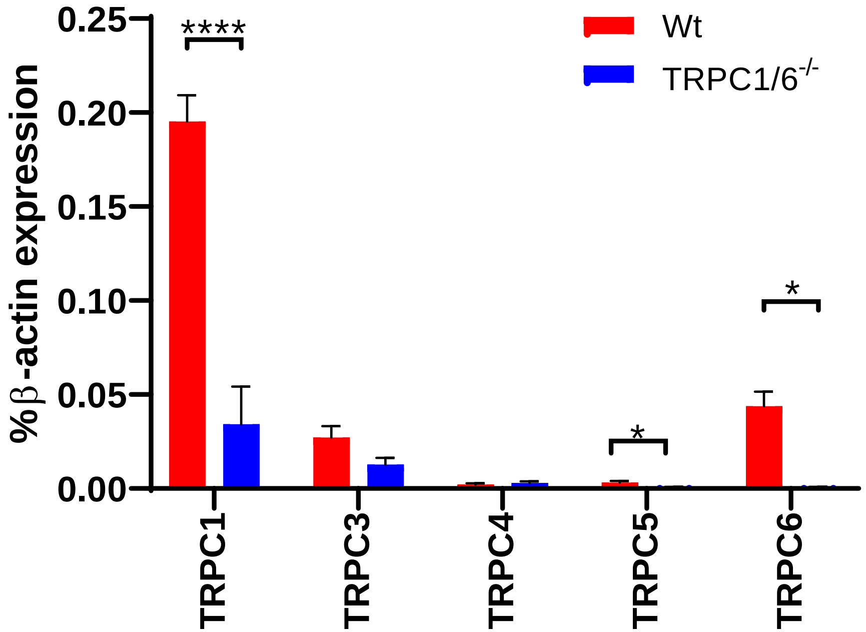

Supplementary Figure 1: Relative mRNA amounts of TRPC channels (TRPC1, 3–6) in WT and TRPC6−/− PMLFs analyzed by quantitative RT-PCR (WT, n = 5 mice; TRPC6−/− n = 5 mice).

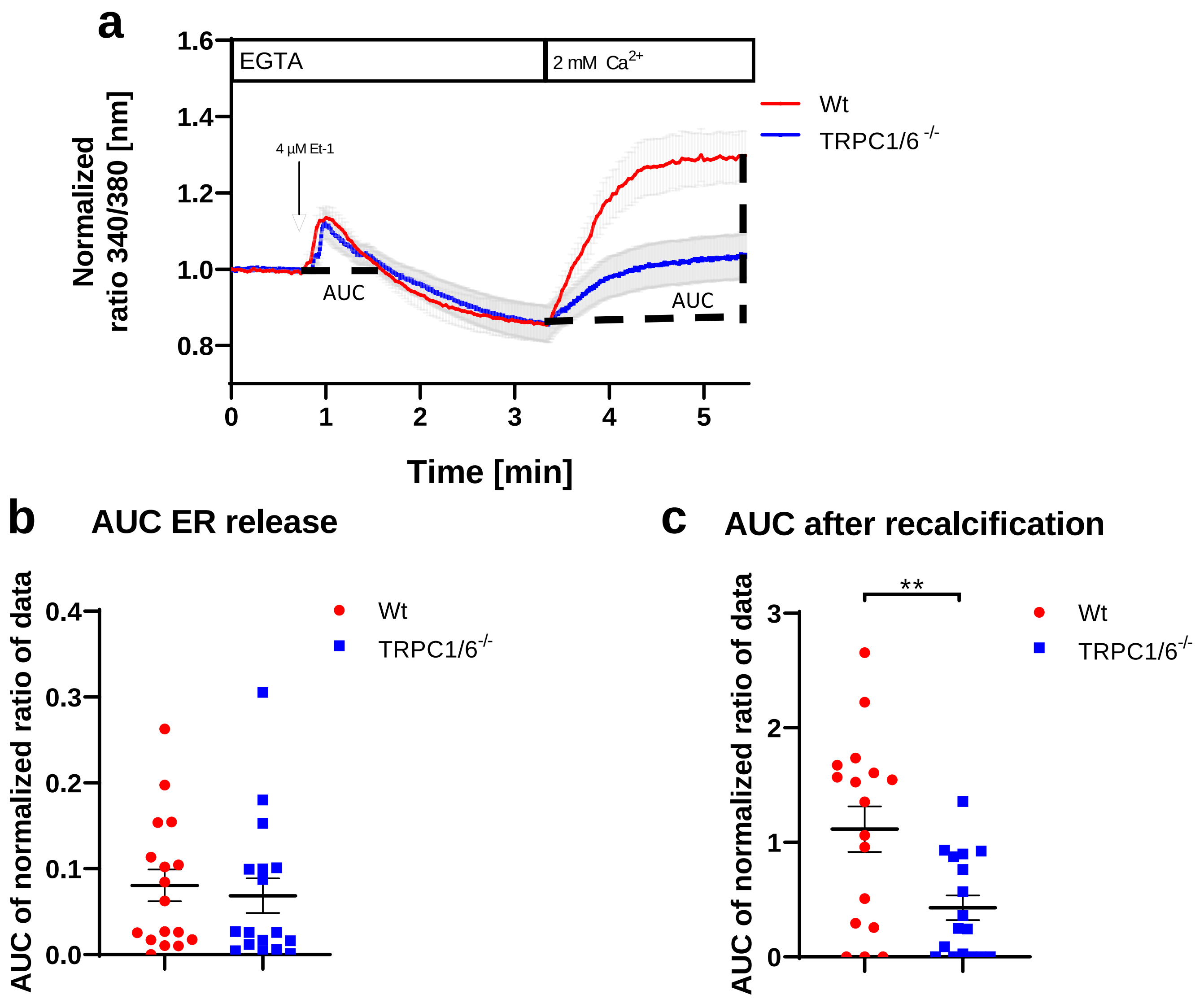

Supplementary Figure 2: Receptor-operated Ca^{2+} entry (ROCE) induced by application of endothelin-1 (Et-1) in Ca^{2+} free medium and after recalcification in TRPC1/6-(TRPC1/6$^{-/-}$) deficient primary murine lung fibroblasts (pmLF). Wild-type (Wt) pmLF served as controls. Fura-2-loaded pmLF were stimulated with 4 µM Et-1 in Ca $^{2+}$ free, EGTA (1.5 mM) containing buffer to empty ER Ca^{2+} stores and Ca^{2+} (2mM) was added to generate ROCE (**a**). Intracellular Ca 2+ levels ([Ca2+]i) were quantified by analysis of fluorescence ratios at excitation wavelengths of 340 and 380 nm (ratio 340/380 nm) and normalized to initial values. Lines represent calculated means and light grey areas indicate standard error of the mean (SEM) of more than three independent experiments of at least three mice. Calculation of the areas under the curves (AUC) in a was used to quantify ROCE (c). One single dot represents the mean of at least 20 cells from one cell isolation. Asterisks mark significant differences from left to right (n = 5 mice, ** $P < 0.01$) between ratios of deficient cells compared to control cells.
