# Supplementary material for: Store-operated Ca2+ entry in primary murine lung fibroblasts is independent of classical transient receptor potential (TRPC) channels and contributes to cell migration
Source: Sci Rep. 2020 Apr 22;10:6812. doi: 10.1038/s41598-020-63677-2 (PMC7176639; doi:10.1038/s41598-020-63677-2)

Whole uncropped blots from Fig. 3

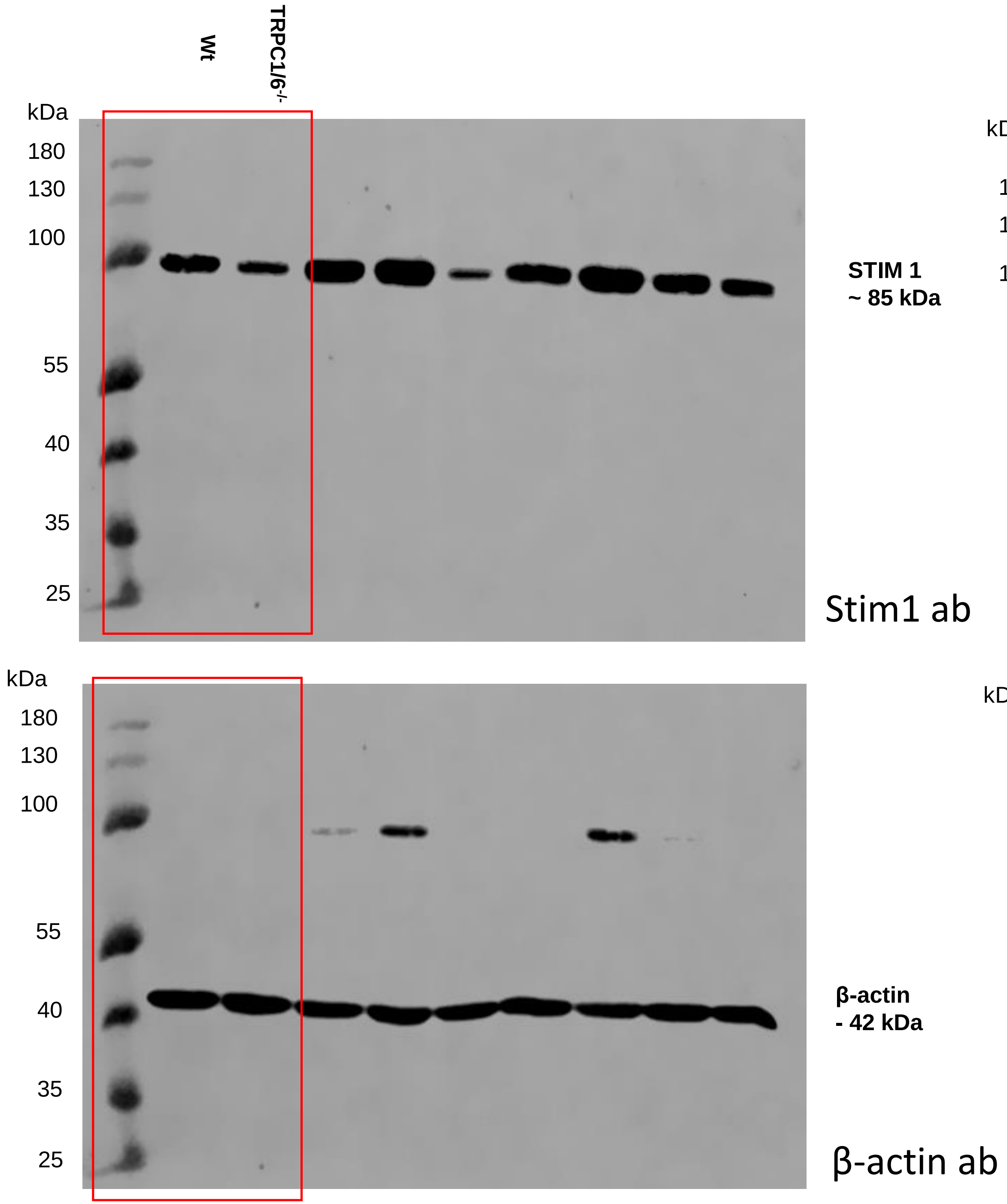

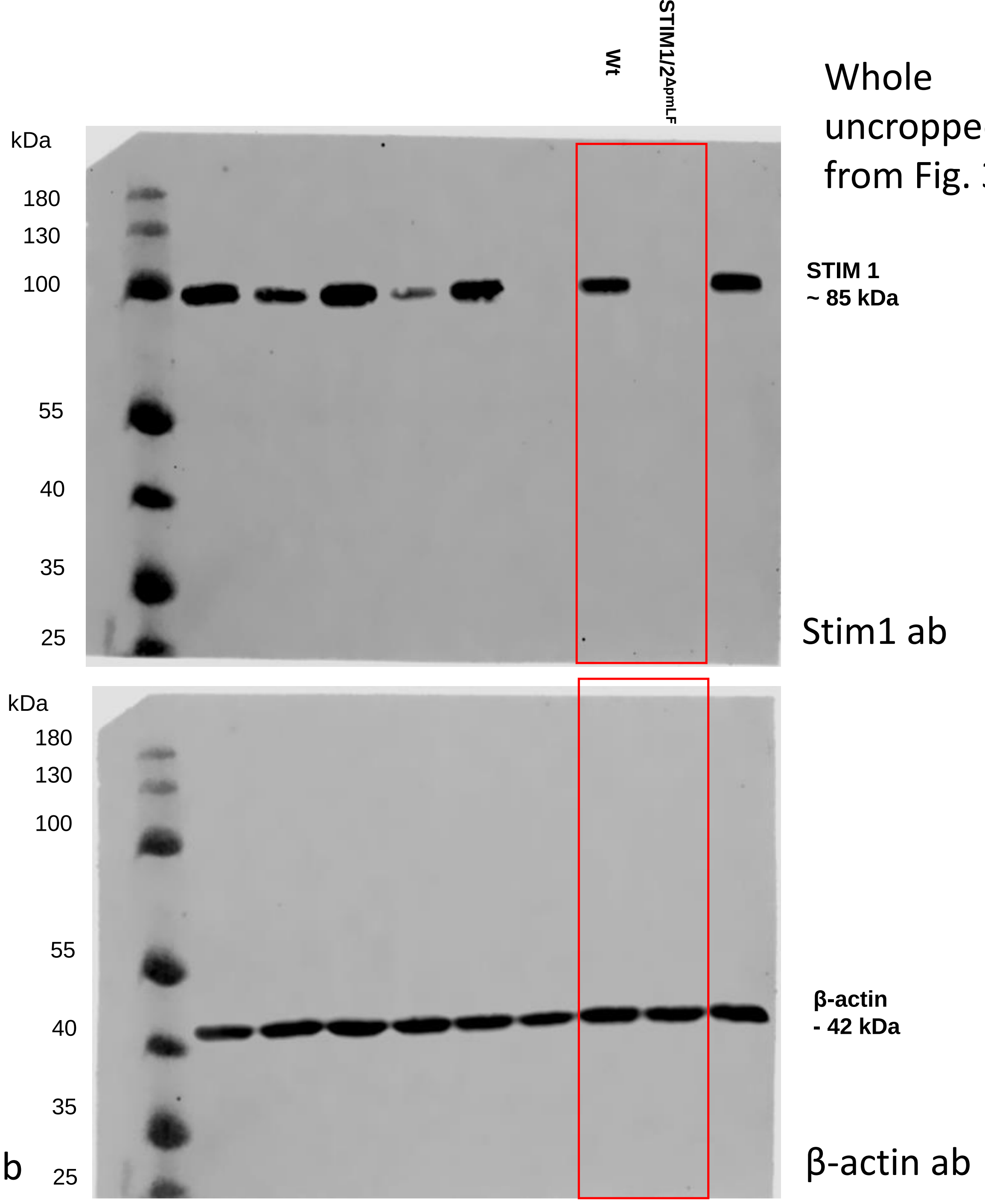

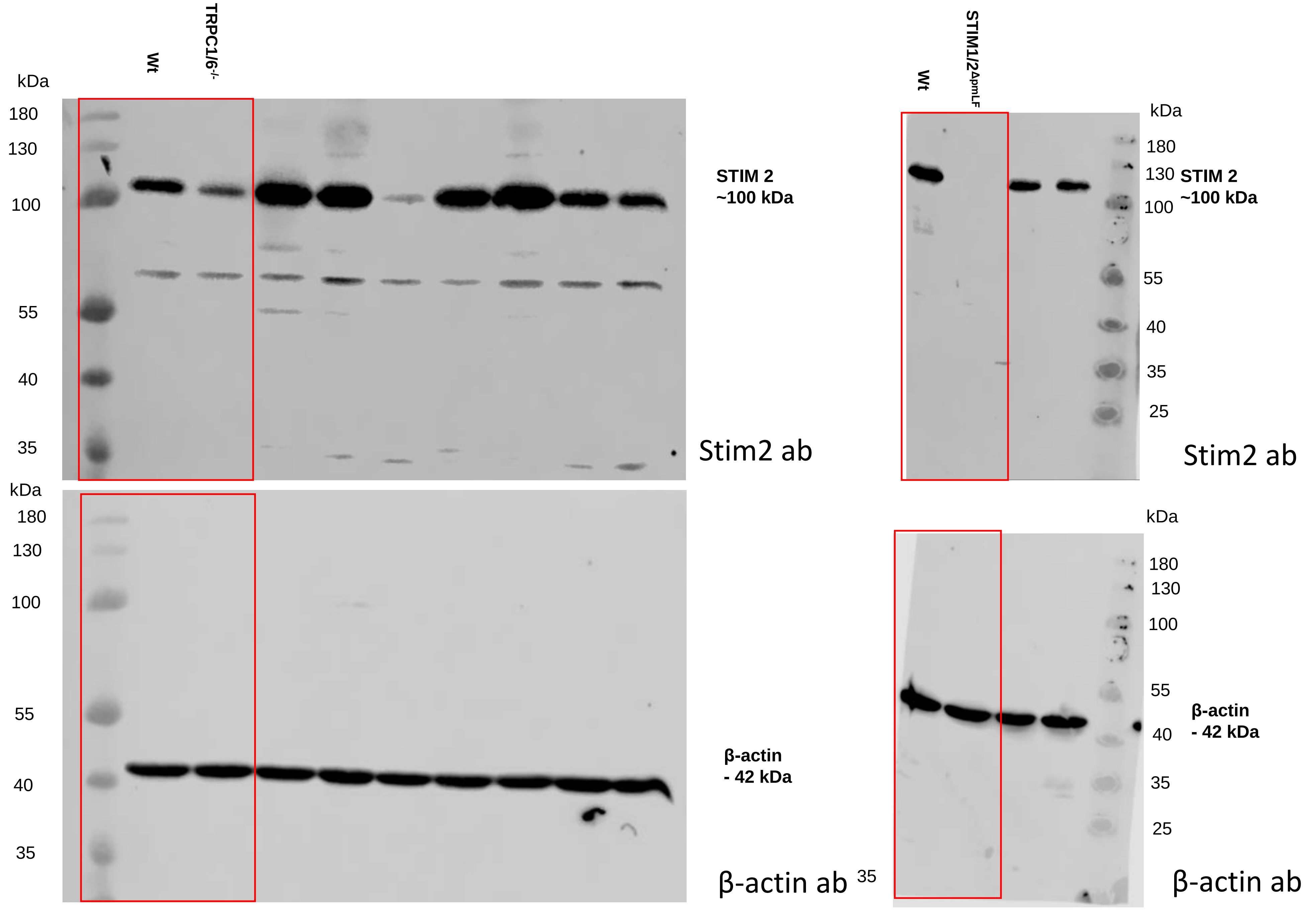

Whole uncropped blots from Fig. 3

Whole uncropped blots from Fig. 8

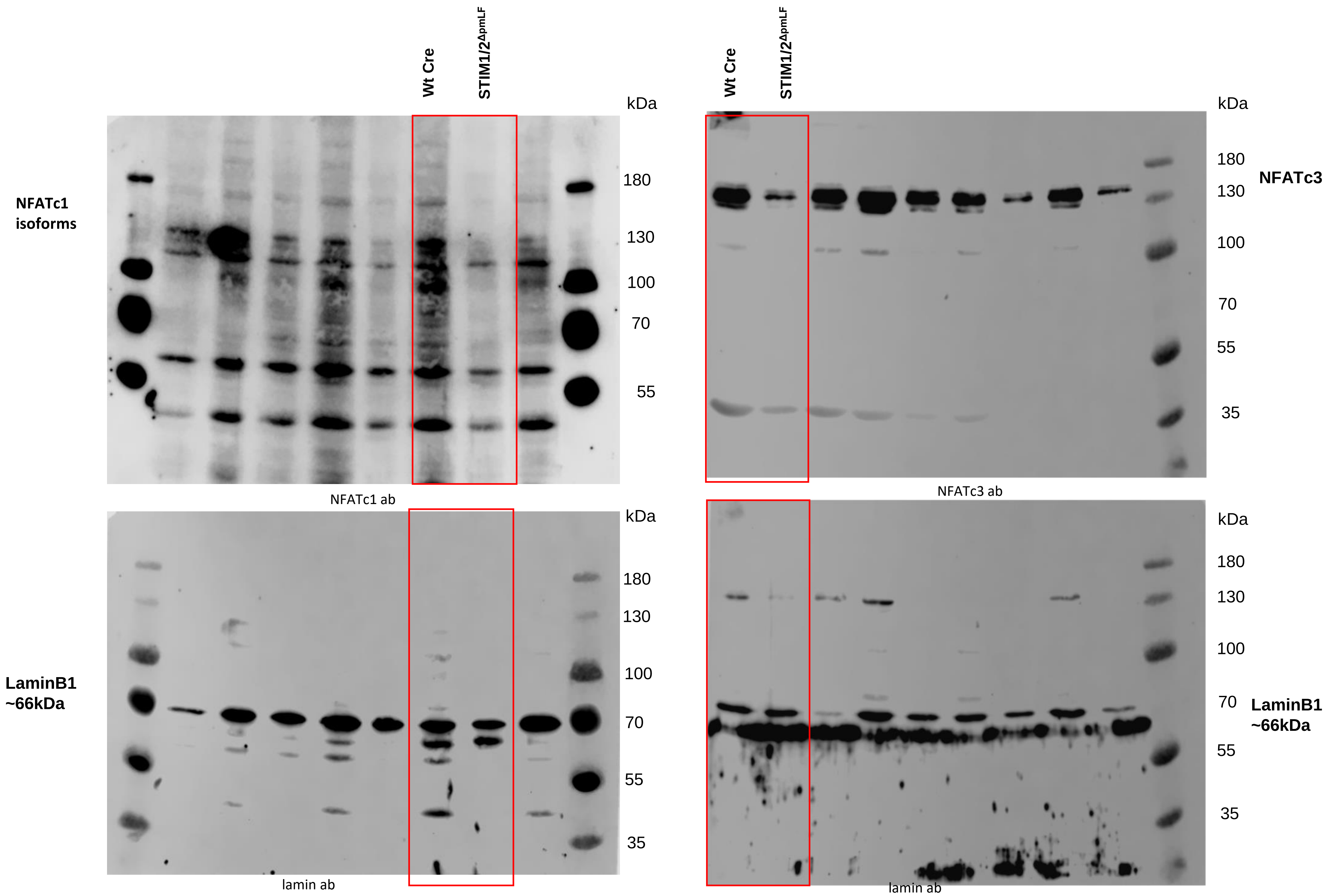

Supplement: Supplementary file 2 — Supplementary Information2. [file 41598_2020_63677_MOESM2_ESM.pdf]
